# Correlation between clinical severity and extent of autonomic cardiovascular impairment in the acute phase of subarachnoid hemorrhage

**DOI:** 10.1007/s00415-022-11220-w

**Published:** 2022-06-20

**Authors:** Matthias C. Borutta, Stefan T. Gerner, Philip Moeser, Philip Hoelter, Tobias Engelhorn, Arnd Doerfler, Hagen B. Huttner, Stefan Schwab, Joji B. Kuramatsu, Julia Koehn

**Affiliations:** 1 Department of Neurology, Friedrich-Alexander-University of Erlangen-Nuremberg (FAU), Schwabachanlage 6, 91054 Erlangen, Germany; 2 Department of Neurology, University of Giessen, Klinikstrasse 33, 35392 Giessen, Germany; 3 Department of Neuroradiology, Friedrich-Alexander-University of Erlangen-Nuremberg (FAU), Schwabachanlage 6, 91054 Erlangen, Germany

**Keywords:** Subarachnoid hemorrhage, Autonomic nervous system, Hunt and Hess, Heart rate variability

## Abstract

**Background and aim:**

To assess associations between clinical severity and possible dysfunction of autonomic cardiovascular modulation within the acute phase after spontaneous subarachnoid hemorrhage (SAH).

**Methods:**

In this prospective observational study, in 51 patients with spontaneous SAH, Hunt-and-Hess scores (H&H) were assessed and cardiovascular autonomic modulation was monitored within 24 h after SAH-onset. From 5 min time-series of R–R-intervals (RRI) and blood-pressure (BP) recordings, we calculated autonomic parameters including time-domain [RRI-coefficient-of-variation (RRI-CV) and square-root-of-the-mean-squared-differences-of-successive-RRIs (RMSSD)] and frequency-domain parameters [low- and high-frequency-powers of RRI- and BP-modulation (RRI-LF-, RRI-HF-, SBP-LF-powers) and RRI-total-powers]. Data were compared to those of 20 healthy volunteers.

**Results:**

RRI- and BP-values did not differ between groups. Yet, parameters of sympathetic (RRI-LF-powers 141.0 (18.9–402.4) ms^2^ vs 442.3 (246.8–921.2) ms^2^, *p* = 0.001) and total autonomic modulation (RRI-CV 2.4 (1.2–3.7) ms^2^ vs 3.7 (3.1–5.3) ms^2^, *p* = 0.001) were significantly lower in patients than in controls. Subgroup analyses (patients with H&H < 3 vs H&H ≥ 3) and Spearman-rank-correlations revealed increasing loss of sympathetic (RRI-LF-powers 338.6 (179.7–710.4) ms^2^ vs 72.1 (10.1–175.9) ms^2^, *p* = 0.001, rho = − 0.524) and total autonomic modulation (RRI-CV 3.5 (2.3–5.4) ms^2^ vs 1.6 (1.0–2.8) ms^2^, *p* < 0.001, rho = − 0.519) with higher H&H-scores. Multiple-logistic-regression underlined the significant influence of H&H-scores on sympathetic (RRI-LF-powers, *p* = 0.033) and total autonomic modulation (RRI-CV, *p* = 0.040) compared to possible confounders (e.g., age, intubation).

**Conclusion:**

Within the acute phase, spontaneous SAH induces a decrease in sympathetic and total autonomic cardiovascular modulation. Higher H&H-scores were associated with increasing autonomic dysfunction and may therefore augment the risk of cardiovascular complications and poor clinical outcome.

**Supplementary Information:**

The online version contains supplementary material available at 10.1007/s00415-022-11220-w.

## Introduction

Non-traumatic subarachnoid hemorrhage (SAH) represents a significant cause of morbidity and mortality throughout the world [1–3]. Although neurological complications, e.g., re-bleeding, hydrocephalus, seizures, and delayed cerebral ischemia, represent relevant aspects that may lead to death and disability after spontaneous SAH, systemic complications may also negatively affect functional outcome [1, 2]. Systemic complications include electrocardio-graphic changes, troponin elevation, neurogenic stunned myocardium, pulmonary edema, anaemia, or hyponatraemia—i.e., conditions that have been associated with a systemic catecholamine release and sympathetic nervous system activation [1, 2].

In several neurological diseases with focal parenchymal injury, e.g., traumatic brain injury (TBI), ischemic stroke, or intracranial haemorrhage, previous studies demonstrated autonomic cardiovascular dysfunction with a decline of overall autonomic modulation and a shift towards sympathetic dominance [4]. So far, only a few studies assessed cardiovascular autonomic modulation after SAH. However, these studies reported contradictory results and only assessed changes of sympathetic and vagal cardiac modulation and heart rate variability (HRV) within the first few days after SAH. Hence, there is uncertainty on initial trauma-induced changes of autonomic cardiovascular modulation compared to healthy persons. Furthermore, there are no studies assessing possible associations between clinical impairment and cardiovascular modulation within the acute phase of the disease as a potentially important marker affecting morbidity and mortality.

We hypothesize that the degree of acute clinical deficits is associated with an increasing risk of autonomic cardiovascular dysfunction. Therefore, we prospectively assessed cardiovascular autonomic modulation within 24 h after spontaneous SAH compared to healthy volunteers, and determined correlations between parameters of autonomic modulation and Hunt-and-Hess scores (H&H) in spontaneous SAH patients.

## Methods

### Patient selection

**Fig. 1:**
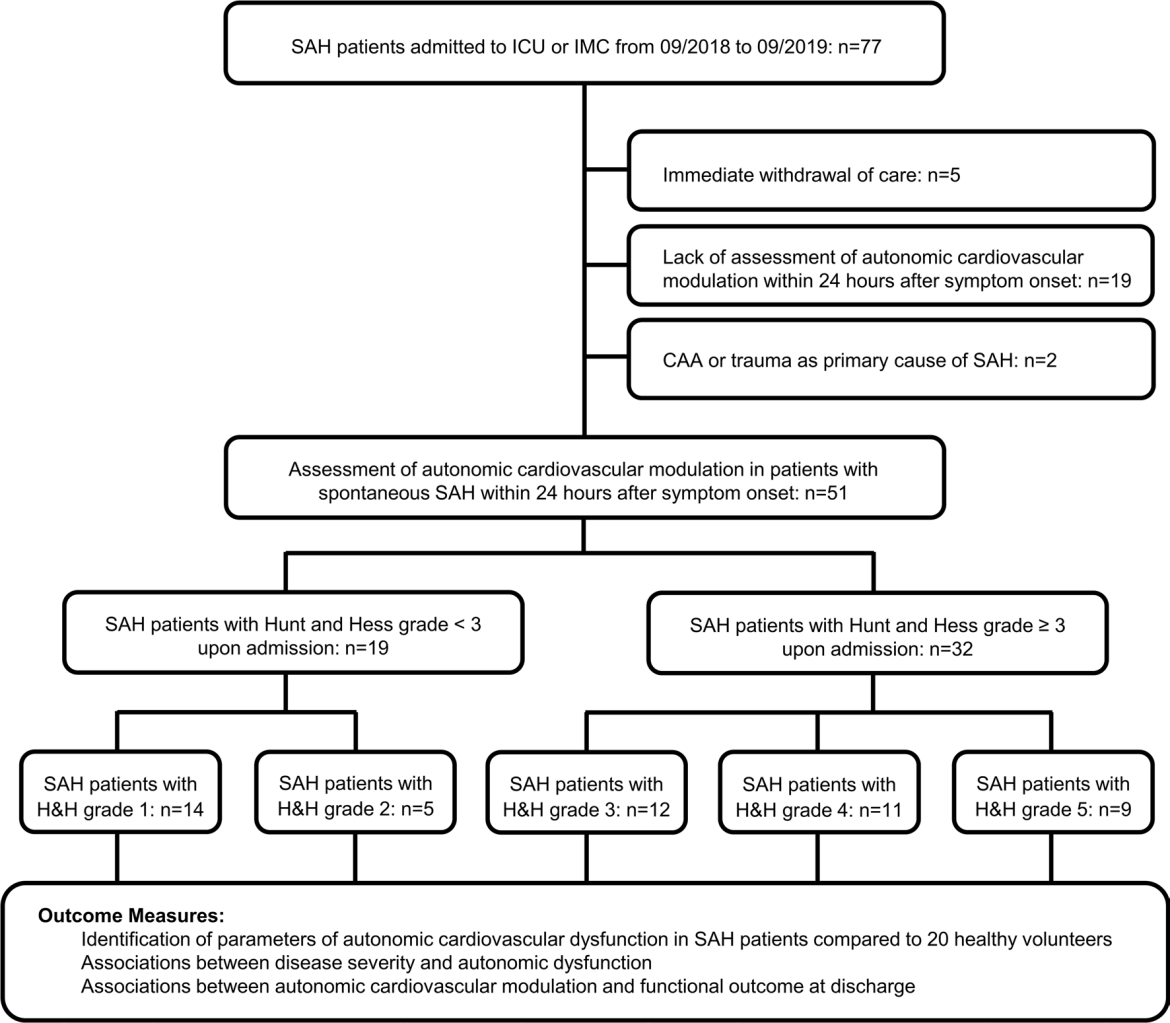
Flow chart of study participants

All patients with SAH admitted to the neuro-emergency department of the Friedrich-Alexander-University Hospital Erlangen-Nuremberg (FAU), Germany, between 09/2018 and 09/2019 were screened for eligibility to participate in the present study. Inclusion criteria consisted of (i) absence of trauma or cerebral amyloid angiopathy as primary cause of SAH, (ii) perimesencephalic SAH, (iii) in-hospital treatment on IMC or neuro-ICU, and (iv) autonomic testing within 24 h after symptom onset (Fig. 1). The study was approved by the ethics committee of the University of Erlangen-Nuremberg, and written informed consent has been obtained from all study participants or their legal representatives according to the Declaration of Helsinki.

### Clinical and radiological parameters

Upon hospital admission, H&H for acute clinical grading (ranging from 1 to 5 with a higher score indicating more severe clinical condition) and the pre-stroke modified Rankin Scale (pre-mRS; ranging from 0 to 5, grading patient’s disability prior to the incident) were assessed. We retrieved data on demographic parameters, prior comorbidities, as well as relevant premedication from the institutional electronic databases. Moreover, we collected data from the initial emergency therapy, i.e., usage of analgesic and sedative drugs, catecholamines, intubation and mechanical ventilation, or CSF drainage via EVD, that patients received before or during autonomic measurement (Table 1 and Suppl-Table 1).

**Table 1:** Baseline and clinical characteristics of 51 patients with spontaneous subarachnoid hemorrhage according to clinical disease severity, either Hunt-and-Hess score < 3 or ≥ 3

Parameter	SAH patients (*n* = 51)	Hunt & Hess < 3 (*n* = 19)	Hunt & Hess ≥ 3 (*n* = 32)	*p* value
Age in years, median (IQR)	54 (50–63)	50 (43–57)	58.5 (51.5–66.5)	***0.006***^**a**^
Women, *n* (%)	27 (52.9%)	8 (42.1%)	19 (59.4%)	0.232
Prior comorbidities, *n* (%)				
Arterial hypertension	26 (51%)	8 (42.1%)	18 (56%)	0.329
Diabetes mellitus	8 (15.7%)	3 (15.8%)	5 (15.6%)	0.640
Dyslipidemia	8 (15.7%)	3 (15.8%)	5 (15.6%)	0.640
Prior ischemic stroke/TIA	1 (2%)	0 (0%)	1 (3.1%)	0.627
Status post endovascular treatment	1 (2%)	0 (0%)	1 (3.1%)	0.627
Congestive heart failure	4 (7.8%)	1 (5.3%)	3 (9.4%)	0.521
Smoker	10 (19.6%)	4 (21.1%)	6 (18.8%)	0.557
Alcohol abuse	3 (5.9%)	1 (5.3%)	2 (6.3%)	0.691
Premedication, *n* (%)				
Antiplatelet drug (ASA, clopidogrel)	6 (11.8%)	2 (10.5%)	4 (12.5%)	0,604
Oral anticoagulant (phenprocoumon)	2 (3.9%)	0 (0%)	2 (6.3%)	0,523
Antihypertensive drug	16 (31.4%)	5 (26.3%)	11 (34.4%)	0,549
Pre-mRS, median (Range)	0 (3)	0 (0)	0 (3)	***0.030***^**a**^
Admission status, median (IQR)				
Heart rate, min^−1^	66.6 (60.1–80.9)	71.7 (64.4–77.7)	64.8 (57.3–81.4)	0.349
Systolic blood pressure, mmHg	130.1 (116.2–144.5)	138.8 (120.4–155.8)	127.2 (111.7–142.5)	0.06
Parameters of SAH on CT scan, *n* (%)				
Detection of SAH on CT scan	50 (98%)	18 (94.7%)	32 (100%)	0.373
Detection of SAH only in CSF	1 (2%)	1 (5.3%)	0 (0%)	0.373
Detection of aneurysm	37 (72.5%)	12 (63.2%)	25 (78.1%)	0.247
*Anterior circulation*	27 (52.9%)	10 (52.6%)	17 (53.1%)	0.888
Ant. comm. artery (ACOM)	16 (31.4%)	8 (42.1%)	8 (25%)	0.203
Middle cerebral artery (MCA)	5 (9.8%)	1 (5.3%)	4 (12.5%)	0.639
Pericallosal artery	1 (2.0%)	0 (0%)	1 (3.1%)	0.627
Internal carotid artery (ICA)	5 (9.8%)	1 (5.3%)	4 (12.5%)	0.639
*Posterior circulation*	10 (19.6%)	2 (10.5%)	8 (25%)	0.287
Post. comm. artery (PCOM)	4 (7.8%)	1 (5.3%)	3 (9.4%)	0.521
Basilar artery (BA)	3 (5.9%)	0 (0%)	3 (9.4%)	0.285
Ant. inf. cerebellar artery (AICA)	1 (2%)	1 (5.3%)	0 (0%)	0.373
Post. inf. cerebellar artery (PICA)	1 (2%)	0 (0%)	1 (3.1%)	0.627
Vertebral artery (VA)	1 (2%)	0 (0%)	1 (3.1%)	0.627
Other sources of bleeding	3 (5.9%)	1 (5.3%)	2 (6.3%)	0.691
Dysplasia of internal carotid artery (ICA)	1 (2%)	1 (5.3%)	0 (0%)	0.373
Dissection of basilar artery (BA)	1 (2%)	0 (0%)	1 (3.1%)	0.627
Arteriovenous fistula in cervical spine	1 (2%)	0 (0%)	1 (3.1%)	0.627
Intracerebral hemorrhage	13 (25.5%)	2 (10.5%)	11 (34.4%)	0.056
Left hemisphere	6 (11.8%)	1 (5.3%)	5 (15.6%)	0.392
Right hemisphere	6 (11.8%)	0 (0%)	6 (18.8%)	0.050
Corpus callosum	1 (2%)	1 (5.3%)	0 (0%)	0.331
Intraventricular hemorrhage	30 (58.8%)	6 (31.6%)	24 (75%)	***0.002***^**a**^
Acute occluding hydrocephalus	30 (58.8%)	5 (26.3%)	25 (78.1%)	** < *0.001***^**a**^
Parameters during first measurement, *n* (%)				
Intubated patient	30 (58.8%)	2 (10.5%)	28 (87.5%)	** < *0.001***^**a**^
Catecholamines (norepinephrine)	21 (41.2%)	1 (5.3%)	20 (62.5%)	** < *0.001***^**a**^
Sedation	29 (56.9%)	2 (10.5%)	27 (84.4%)	** < *0.001***^**a**^
External ventricular drain	***29 (56.9%)***	***5 (26.3%)***	***24 (75.0%)***	***0.001***^***a***^
mRS at discharge, Median (IQR)	2 (0–5)	0 (0–1)	4 (1–5)	** < *0.001***^**a**^

*SAH* subarachnoid hemorrhage, *n* number, *IQR* interquartile range, *TIA* transient ischemic attack, *ASA* acetylsalicylic acid, *SD* standard deviation, *pre-mRS* modified Rankin Scale before ictus, *min*^*−1*^ per minute, *mmHg* millimeter of mercury, *CT* computed tomography, *CSF* cerebrospinal fluid, *mRS* modified Ranking scale

^a^Significant differences between subgroups

Diagnosis of SAH was made upon cranial computed tomography (CT) imaging (Somatom Definition AS +) or spinal tap (xanthochromia and more than 2000 red blood cells/µl) [5]. CT-scans were reviewed by three neuroradiologists blinded to clinical parameters. Investigators documented concomitant intracerebral hemorrhage, intraventricular hemorrhage (IVH), and acute hydrocephalus (enlargement of lateral ventricles measured as bicaudate index > 95th percentile for age; Table 1) [6]. The decision to perform digital subtraction angiography for detection of the source of bleeding followed by endovascular or surgical treatment of aneurysm if applicable was made by the treating physician.

Upon hospital discharge, clinical outcome, i.e., mRS, as a functional parameter for disability was assessed (ranging from 0 to 6, with values of 0–3 indicating favourable outcome and 4–6 indicating unfavourable outcome; Table 1).

### Parameters of autonomic cardiovascular modulation

Within 24 h after symptom onset, parameters of cardiovascular autonomic modulation were monitored via 3-lead electrocardiography (sampled at 200 Hz) for 5 min time-series of R–R-intervals (RRI) and beat-to-beat systolic and diastolic blood pressure (SBP, DBP) with non-invasive measurement via finger pulse photoplethysmography (Portapres^®^, TPD Biomedical Instrumentation, Amsterdam, The Netherlands) at the index or middle finger [7]. The blood pressure (BP) was calibrated against the ipsilateral brachial artery BP [7]. Analysis of the digitized data was performed on a custom-designed data acquisition and analysis system (SUEmpathy™, SUESS Medizin-Technik GmbH, Aue, Germany) [8].

Within the 5 min recordings, the most stationary 2 min-segments (i.e. clear signals without arrhythmias) were extracted followed by manual correction of artefacts and calculation of the mean values and standard deviation (SD) of all signs.

From the 2 min segments, time-domain parameters of cardiovascular autonomic modulation were calculated, i.e., the coefficient of variation of RRIs (RRI-CV) and the RRI-SD both reflecting sympathetic and vagal cardiac modulation as well as square root of the mean squared differences of successive RRIs (RMSSD) which is assumed to uniquely reflect vagal cardiac modulation [4, 9, 10].

RRI- and BP-values show slow underlying fluctuations that are largely mediated by undulating activity of the autonomic nervous system (ANS) [11]. We performed spectral analysis of slow sympathetically and parasympathetically mediated RRI and BP oscillations, using trigonometric regressive spectral analysis (TRS) algorithm [7]. The TRS algorithm identifies two main peaks of oscillation of RRI and BP modulation: the low-frequency (LF; 0.04–0.14 Hz) and high-frequency (HF; 0.15–0.50 Hz) range [4, 9, 10]. The magnitude of LF and HF oscillations can be calculated as integral under the power spectral density curves of RRI (ms^2^/Hz) and BP (mmHg^2^/Hz), and is then defined as LF- and HF-powers of RRI (ms^2^) and BP (mmHg^2^) [4, 9, 10].

LF oscillations of RRI at rest (RRI-LF-powers) are described to be influenced by sympathetic outflow and also, to an uncertain degree, by parasympathetic outflow [4, 9, 10]. On contrary, LF oscillations of BP (SBP-LF-powers) are solely depending upon sympathetic outflow [4, 9, 10]. HF oscillations of RRI (RRI-HF-powers) are related to parasympathetic outflow, whereas fluctuations in the HF range of BP are mainly caused by respiration-induced fluctuations in venous return and cardiac output [4, 9, 10].

As an additional parameter for total autonomic cardiac modulation, we determined total powers of RRI oscillations (RRI-total powers) as sum of LF- and HF-powers. Finally, RRI-LF/HF-ratios were calculated as an index for sympatho-vagal balance [4, 9, 10].

Cardiovascular autonomic parameters of patients were compared to data of 20 healthy volunteers with no prior cardiovascular diseases or medication. Healthy controls were tested between 9AM and 2PM in supine position in a quiet room with ambient temperature of approximately 24 °C and stable humidity. Before testing, participants initially rested for 40 min to ensure a stable cardiovascular situation.

### Statistical analysis

For data analysis, we used a commercially available statistical program (IBM SPSS Statistics for Windows, version 24). Significance was set at *p* < 0.05. Testing for normal distribution was performed using the Kolmogorov–Smirnov test. Data are expressed as median and interquartile range. Normally distributed patient and control data were compared using the *t* test for unpaired samples and the Mann–Whitney-*U* test for comparison of non-normally distributed data.

For subgroup analysis, we categorized patients according to their clinical condition into two groups: H&H-scores 1–2 (H&H < 3; no neurological deficit other than cranial nerve palsy) and H&H-scores 3–5 (H&H ≥ 3; altered level of consciousness and neurological deficits). As age-dependant normative values had only been determined for time-domain parameters, we chose RRI-CV as a parameter of total autonomic cardiovascular modulation to further dichotomize our patient cohort [10]. Therefore, another subgroup analysis was carried out by assessing associations between normal vs pathological RRI-CV and the mRS at discharge, Fig. 3).

**Fig. 2:**
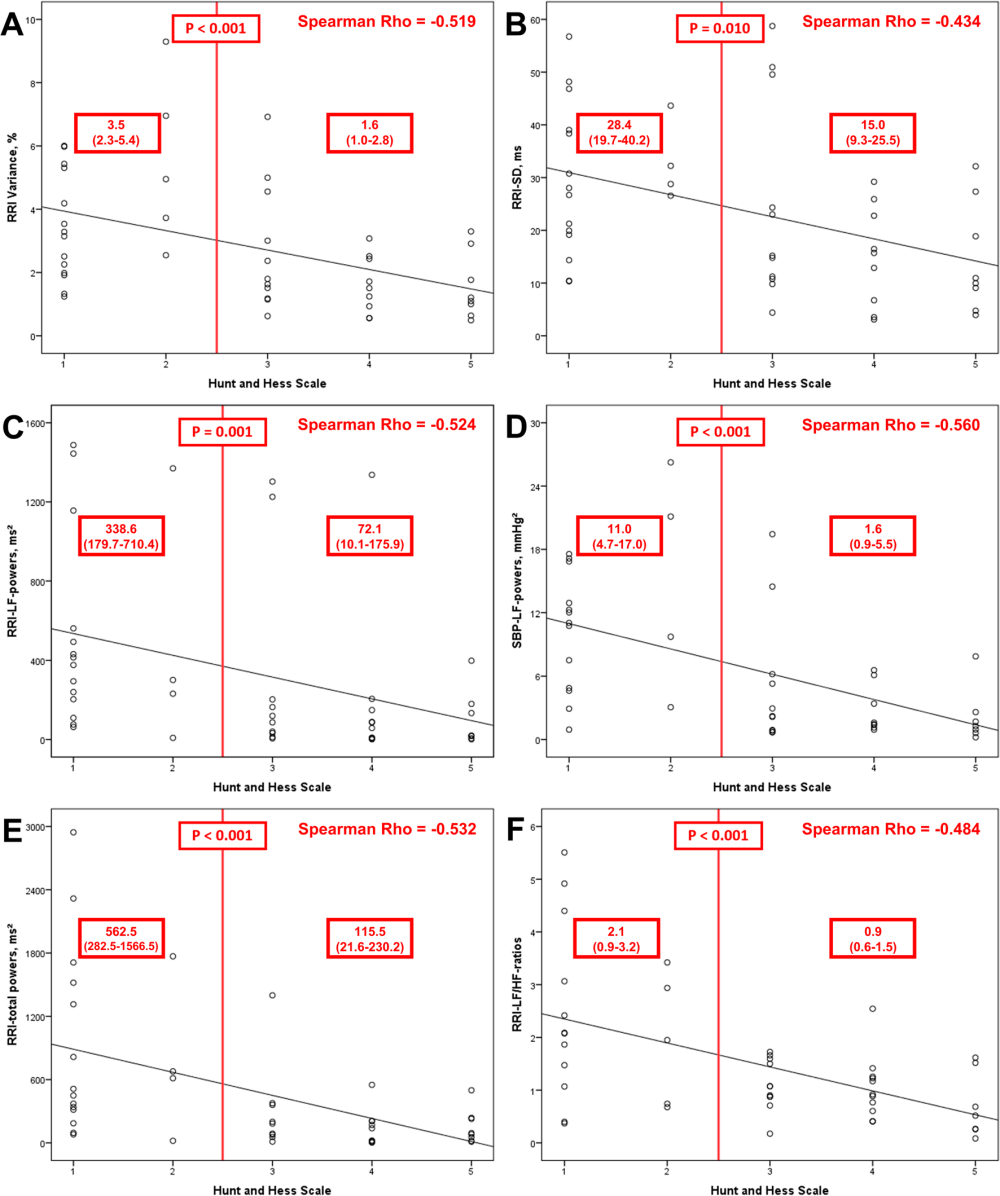
Prognostic performance of autonomic cardiovascular parameters for identification of patients with poor clinical outcome

Correlations between H&H-scores, all bio-signals as well as autonomic cardiovascular parameters were assessed using Spearman rank correlation. Receiver-operating-characteristic (ROC) analysis was performed to investigate associations of H&H-scores with autonomic cardiovascular modulation and to determine the best cut-off values for prediction of H&H ≥ 3. To adjust for possible baseline confounders, multivariate analyses (binary logistic regression) were adjusted for established parameters—age, pre-mRS, IVH, and intubation—i.e., parameters identified to be associated with initial clinical severity according to H&H-scores in univariate analyses (Table 1).

## Results

Over a 12 month period, a total of 77 patients with SAH were screened for eligibility (Fig. 1). After exclusion of patients with immediate withdrawal of care due to unfavourable prognosis, lack of assessment of autonomic cardiovascular modulation within 24 h after symptom onset, and cerebral Amyloid angiopathy or trauma as primary cause of SAH, 51 patients (H&H < 3: *n* = 19, H&H ≥ 3: *n* = 32) were enrolled in the study (27 women, 24 men; 54.0 (50.0–63.0) years). Clinical baseline characteristics of patients and controls are presented in Tables 1 and 2. Patients with higher H&H-scores were significantly older than patients with lower H&H-scores (*p* = 0.005), as well as controls (*p* < 0.001). Although patients with higher H&H-scores had a slightly though significantly higher pre-mRS than patients with lower H&H-scores [Median (Range), 0 (3) vs 0 (0); *p* = 0.030], both groups did not differ regarding prior cardio- and cerebrovascular comorbidities and premedication (Table 1, Suppl-Table 1). Most radiological parameters of SAH on admission CT-scans did not differ between both groups, yet patients with higher H&H-scores had higher incidences of occluding hydrocephalus, concomitant parenchymal, and intraventricular hemorrhage. Moreover, during the first 24 h after symptom onset, acute intensive care interventions (i.e., mechanical ventilation, use of analgo-sedation, vasopressor therapy, or CSF drainage via EVD) were significantly more often required in patients with higher H&H-scores (Table 1).

**Table 2:** Biosignals and parameters of autonomic cardiovascular modulation of 51 SAH patients, Hunt & Hess < 3 (*n* = 20) and Hunt & Hess ≥ 3 (*n* = 19) and 20 healthy controls

	SAH patients	Healthy controls	H&H < 3	H&H ≥ 3	*p* value	*p* value	*p* value	*p* value
Parameter, median (IQR)	(*n* = 51)	(*n* = 20)	(*n* = 19)	(*n* = 32)	Pat vs Ct	H&H < 3 vs Ct	H&H** ≥ **3 vs Ct	H&H < 3 vs H&H** ≥ **3
Age, years	54.0 (50.0–63.0)	50.0 (37.5–55.8)	50.0 (43.0–57.0)	58.5 (51.5–66.5)	***0.002***^**b, c**^	0.258^c^	** < *0.001***^**a, b**^	***0.005***^**b, c**^
Sex (women), *n* (%)	27 (52.9%)	11 (55.0%)	8 (42.1%)	19 (59.4%)	0.888	0.420	0.752	0.232
Biosignals								
RRI mean, ms	886.0 (739.3–995.7)	754.1 (728.7–926.9)	859.0 (752.0–940.1)	908.1 (735.1–1042.8)	0.185^a^	0.706^c^	0.181^c^	0.349^c^
SBP mean, mmHg	130.1 (116.2–144.5)	128.4 (122.2–139.0)	138.8 (120.4–155.8)	127.2 (111.7–142.5)	0.347^c^	0.081^c^	0.853^c^	0.060^c^
Parameters of sympathetic modulation								
RRI-LF-powers, ms^2^	141.0 (18.9–402.4)	442.3 (246.8–921.2)	338.6 (179.7–710.4)	72.1 (10.1–175.9)	***0.001***^**a, b**^	0.317^c^	** < *0.001***^**a, b**^	***0.001***^**a, b**^
SBP-LF-powers, mmHg^2^	3.4 (1.3–11.0)	14.5 (7.8–20.4)	11.0 (4.7–17.0)	1.6 (0.9–5.5)	** < *0.001***^**a, b**^	0.131^c^	** < *0.001***^**a, b**^	** < *0.001***^**a, b**^
Parameters of parasympathetic modulation								
RRI-RMSSD, ms	17.9 (11.8–36.5)	20.6 (14.2–28.3)	26.0 (14.7–40.1)	15.9 (9.9–30.2)	0.279^c^	0.103^c^	0.856^c^	0.171^c^
RRI-HF-powers, ms^2^	77.2 (18.7–128.8)	145.3 (32.0–258.2)	97.7 (19.1–311.9)	73.8 (13.1–103.9)	0.114^c^	0.710^c^	***0.041***^**b, c**^	0.151^c^
Parameters of total autonomic modulation								
RRI-total powers, ms^2^	207.1 (78.4–540.7)	624.0 (353.9–1588.6)	562.5 (282.5–1566.5)	115.5 (21.6–230.2)	***0.001***^**a, b**^	0.608^c^	** < *0.001***^**a, b**^	** < *0.001***^**a, b**^
RRI-SD, ms	20.6 (10.7–31.1)	30.4 (23.2–45.9)	28.4 (19.7–40.2)	15.0 (9.3–25.5)	***0.011***^**a, b**^	0.530^c^	***0.001***^**a, b**^	***0.010***^**b, c**^
RRI-CV, %	2.4 (1.2–3.7)	3.7 (3.1–5.3)	3.5 (2.3–5.4)	1.6 (1.0–2.8)	***0.002***^**a, b**^	0.499^c^	** < *0.001***^**a, b**^	** < *0.001***^**a, b**^
Index of sympatho-vagal-balance								
RRI-LF/HF-ratios	1.1 (0.7–1.9)	4.0 (1.7–10.0)	2.1 (0.9–3.2)	0.9 (0.6–1.5)	** < *0.001***^**a, b**^	***0.005***^**b, c**^	** < *0.001***^**a, b**^	***0.003***^**a, b**^

*SAH* subarachnoid hemorrhage, *n* number, *H&H* Hunt-and-Hess score, *Pat* patients, *Ct* healthy controls, *RRI* R–R interval, *SBP* systolic blood pressure, *LF* low frequency, *HF* high frequency, *RMSSD* root mean square of successive differences, *ms* milliseconds, *mmHg* millimeter of mercury, *SD* standard deviation, *CV* coefficient of variance

^a^*p* values derived from the nonparametric Mann–Whitney-test

^b^significant differences between patients and controls

^c^*p* values derived from *t* tests

### Parameters of cardiovascular autonomic modulation: SAH patients vs controls

Overall, between SAH patients and controls, there were no significant differences in the assessed bio-signals used for further analysis of autonomic modulation, i.e., RRIs and SBP (Table 2). Parameters reflecting sympathetic cardiac modulation, i.e., RRI-LF-powers and SBP-LF-powers were significantly lower in SAH patients than in controls. Similarly, patients had significantly lower values of parameters of total autonomic modulation, i.e., RRI-total powers, RRI-SD and RRI-CV, as well as RRI-LF/HF-ratios as an index of sympatho-vagal balance (Table 2). In contrast, parameters of vagal cardiac modulation did not differ between patients and controls.

### Subgroup analyses of parameters of cardiovascular autonomic modulation: patients with H&H < 3 vs controls and patients with H&H ≥ 3 vs controls

In a next step, we compared parameters of cardiovascular autonomic modulation between patients with lower and higher H&H-scores to those of healthy controls. While parameters reflecting sympathetic (RRI-LF-powers, SBP-LF-powers), parasympathetic (RRI-HF-powers), and total autonomic modulation (RRI-total powers, RRI-SD, RRI-CV) only showed a trend towards lower values in patients with lower H&H-scores compared to controls, these differences reached statistical significance when comparing patients with higher H&H-scores to healthy controls (Table 2). Only RRI-LF/HF-ratios as an index of sympatho-vagal-balance were significantly lower in both patient subgroups than in controls (Table 2).

### Subgroup analyses of parameters of cardiovascular autonomic modulation: patients with H&H < 3 vs H&H ≥ 3

To investigate possible associations between cardiovascular autonomic modulation and clinical SAH severity, we compared parameters between patients with lower and higher H&H-scores (Table 2). With increasing SAH severity, subgroup analyses revealed a decrease of parameters reflecting sympathetic cardiac modulation (RRI-LF-powers, SBP-LF-powers), total autonomic modulation (RRI-total powers, RRI-SD, RRI-CV), as well as sympatho-vagal-balance (RRI-LF/HF-ratios). The parameters RRI-RMSSD and RRI-HF-powers, reflecting vagal cardiovascular outflow, only showed a trend towards lower values in patients with higher compared to patients with lower H&H-scores (*p* > 0.05; Table 2).

### Associations between parameters of cardiovascular autonomic modulation and SAH severity

There was no significant correlation between H&H-scores and RRIs or parameters of vagal cardiac modulation (RRI-RMSSD and RRI-HF-powers). In contrast, SAH severity correlated inversely and significantly with SBP (Rho = − 0.291, *p* = 0.047), indices of sympathetic cardiovascular modulation, i.e., RRI-LF-powers (Rho = − 0.524, *p* < 0.001) and SBP-LF-powers (Rho = − 0.560, *p* < 0.001), total autonomic modulation, i.e., RRI-total powers (Rho = − 0.532, *p* < 0.001), RRI-SD (Rho = − 0.434, *p* = 0.003), and RRI-CV (Rho = − 0.519, *p* < 0.001), as well as sympatho-vagal-balance, i.e., RRI-LF/HF-ratios (Rho = − 0.484, *p* = 0.001; Fig. 2).

In a next step, we calculated cut-off values of autonomic cardiovascular parameters best discriminative for H&H ≥ 3. All parameters of sympathetic modulation, total autonomic modulation, and sympatho-vagal balance showed significant associations with SAH severity [AUC (95%CI): RRI-LF-powers 0.800 (0.666–0.933), *p* = 0.001; SBP-LF-powers 0.846 (0.725–0.967), *p* < 0.001; RRI-total powers 0.825 (0.697–0.952), *p* < 0.001; RRI-SD 0.746 (0.602–0.890), *p* = 0.005; RRI Variance 0.810 (0.688–0.932), *p* < 0.001; RRI-LF/HF-ratios 0.767 (0.604–0.930), *p* = 0.003], while there were no significant associations between parameters of parasympathetic modulation and SAH severity [AUC (95%CI): RRI-HF-powers 0.637 (0.460–0.814), *p* = 0.145; RRI-RMSSD 0.633 (0.470–0.797), *p* = 0.12].

Given possible influences of differences in baseline and clinical characteristics on autonomic cardiovascular modulation, we performed multivariate analyses corrected for confounding variables to verify associations of autonomic cardiovascular dysfunction and clinical SAH severity (Table 3). The exploratory results of these multivariate analyses did not reveal significant associations between autonomic cardiovascular modulation and age, pre-mRS, intraventricular hemorrhage, and intubation (*p* > 0.050 for all possible variables confounding the assessed parameters of autonomic cardiovascular modulation).

**Table 3:** Multivariable logistic regression corrected for confounding variables to verify associations between autonomic cardiovascular dysfunction and clinical SAH severity

	*p* value	aOR	95% CI	
RRI-LF-powers [ms^2^]—ROC-analysis AUC (95% CI) for H&H ≥ 3: 0.800 (0.666–0.933), *p* = *0.001*^*a*^				
Multivariable logistic regression model	***0.003***^***a***^			
Age	0.792	0.99	0.92	1.07
Pre-mRS	0.690	1.34	0.32	5.69
Intraventricular hemorrhage	0.755	1.31	0.24	7.00
Intubation	0.538	1.95	0.24	16.14
Hunt-and-Hess score	***0.033***^***a***^	9.20	1.20	70.48
SBP-LF-powers [mmHg^2^]—ROC-analysis AUC (95% CI) for H&H ≥ 3: 0.846 (0.725–0.967), *p* < *0.001*^*a*^				
Multivariable logistic regression model	** < *0.001***^***a***^			
Age	0.845	0.99	0.92	1.07
Pre-mRS	0.067	11.90	0.84	168.23
Intraventricular hemorrhage	0.261	2.95	0.45	19.55
Intubation	0.924	0.87	0.05	15.94
Hunt-and-Hess score	***0.026***^***a***^	36.29	1.55	850.23
RRI-SD [ms]—ROC-Analysis AUC (95% CI) for H&H ≥ 3: 0.746 (0.602–0.890), *p* = *0.005*^*a*^				
Multivariable logistic regression model	***0.015***^***a***^			
Age	0.217	0.96	0.89	1.03
Pre-mRS	0.141	2.91	0.10	12.03
Intraventricular hemorrhage	0.818	0.83	0.16	4.20
Intubation	0.765	0.73	0.09	5.82
Hunt-and-Hess score	***0.027***^***a***^	12.49	1.34	116.65
RRI-CV [%]—ROC-analysis AUC (95% CI) for H&H ≥ 3: 0.810 (0.688–0.932), *p* < *0.001*^*a*^				
Multivariable logistic regression model	***0.001***^***a***^			
Age	0.228	0.95	0.88	1.03
Pre-mRS	0.153	2.84	0.68	11.91
Intraventricular hemorrhage	0.327	0.38	0.06	2.62
Intubation	0.379	2.68	0.30	24.09
Hunt-and-Hess score	***0.040***^***a***^	12.16	1.13	131.50

*p* values for confounding variables and clinical severity according to Hunt-and-Hess score for RRI-HF-powers, RRI-RMSSD, RRI-total powers, and RRI-LF/HF-ratios were not significant (*p* value > 0.050). SAH, subarachnoid hemorrhage

*ROC* receiver-operating characteristics, *AUC* area under the curve, *aOR* adjusted odds-ratio, *CI* confidence interval, *H&H* Hunt-and-Hess score, *Pre-mRS* pre-stroke modified Rankin Scale, *RRI* R-R interval, *SBP* systolic blood pressure, *LF* low frequency, *ms* milliseconds, *mmHg* millimeter of mercury, *SD* standard deviation, *CV* coefficient of variance

^a^Significant correlation

In contrast, we identified clinical SAH severity as independent variable for impaired autonomic sympathetic cardiovascular modulation [RRI-LF: OR 9.20 (1.20–70.48), *p* = 0.033; SBP-LF: OR 47.95 (1.96–1173.02), *p* = 0.018] and parameters of total autonomic cardiovascular modulation [RRI-SD: OR 12.49 (1.34–116.65), *p* = 0.027; RRI-CV: OR 12.16 (1.13–131.50), *p* = 0.040; Table 3].

### Prognostic value of cardiovascular autonomic parameters for clinical outcome in SAH patients

**Fig. 3:**
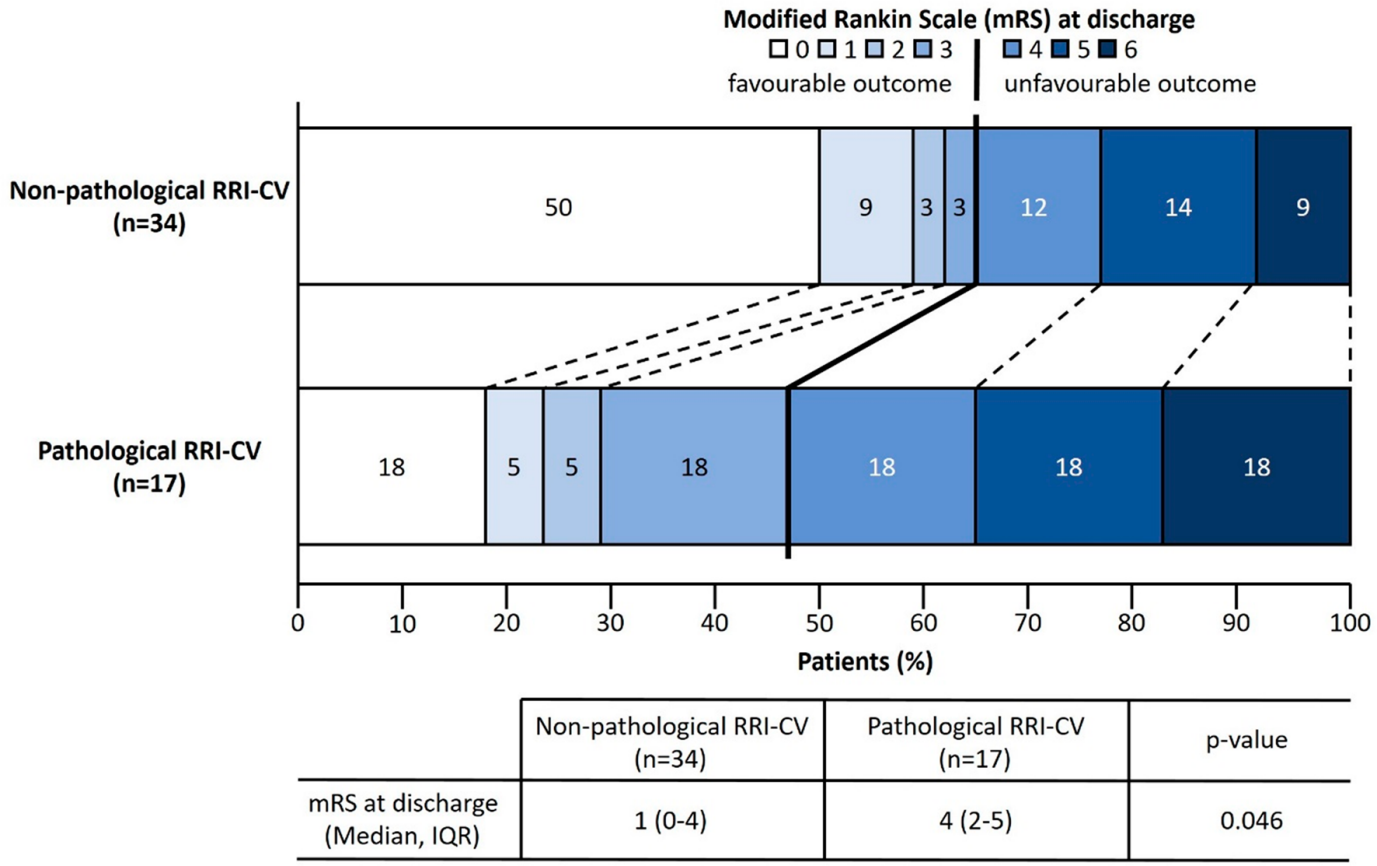
RRI-CV as a parameter of total autonomic cardiovascular modulation in relation to disease severity

To investigate possible associations between parameters of autonomic cardiovascular modulation within 24 h after symptom onset and short-term clinical SAH outcome at discharge, we additionally performed subgroup analyses using RRI-CV as a parameter of total autonomic cardiovascular modulation with established age-dependant normative values. Dichotomisation of our patient cohort into the two groups non-pathological (*n* = 34) vs pathological (*n* = 17) RRI-CV revealed clear associations between impaired autonomic cardiovascular modulation within 24 h after SAH onset and functional outcome at discharge (Fig. 3). Thus, patients with impaired cardiovascular autonomic modulation had significantly higher rates of unfavourable outcome at discharge compared to patients with normal RRI-CV (Fig. 3).

## Discussion

To the best of our knowledge, this is the first observational study prospectively assessing a possible impairment of autonomic cardiovascular modulation in SAH patients in association with clinical severity during the acute phase after symptom onset. In our patients, we were able to demonstrate an inverse correlation between parameters of sympathetic and total autonomic cardiovascular modulation and H&H-scores, i.e., an increasing reduction of autonomic cardiovascular modulation with higher H&H-scores independent of potential confounding variables like age, mechanical ventilation, and co-medication.

In general, previous trials have controversially discussed possible influences of SAH on the central autonomic control. In contrast to our findings in SAH patients, animal trials described an increase in sympathetic activity and BP modulation accompanied by a distinct release of plasma catecholamines within the first hours after experimentally induced SAH [12, 13]. In clinical trials, sympathetic overexcitation has been described within the first few days after ictus and suggested to be responsible for multiple systemic complications like neurogenic pulmonary edema or myocardial contraction band necrosis [2, 14]. In contrast, other authors described a loss of parasympathetic activity within the first few days after SAH and suggested an association between lower values of vagal cardiovascular modulation and life-threatening secondary complications, i.e., cerebral vasospams, delayed cerebral ischemia, and sepsis [15, 16]. Finally, some trials postulated a combined dysfunction with augmented vagal and sympathetic activity within the first days after SAH [17].

Despite these contradictory findings, acute focal parenchymal brain lesions, e.g., due to ischemic stroke especially with insular cortical damage, but also traumatic brain injury, have been postulated to induce a decrease of total autonomic modulation with a shift towards sympathetic predominance [1, 4, 18, 19]. A possible explanation for this may be the different underlying pathophysiology of brain injury caused by these diseases, i.e., a focal parenchymal lesion with a consecutive dysfunction of central autonomic network structures, including supratentorial and infratentorial areas (“brain–heart axis”) [1].

In contrast to observations in patients with focal brain lesions, the decrease of total and sympathetic autonomic modulation of our patients may be explained by a diffuse disruption of autonomic network structures. After SAH, pathophysiological processes induce a rapid endocrine stress response through the hypothalamic–pituitary–adrenal axis [1]. These dynamic changes have been described in animal trials, where sympathoexcitation with increasing levels of serum cortisol, aldosterone, and catecholamines in the hyperacute phase after experimentally induced SAH was observed [12, 13]. Although no data exist for these very early changes in humans, a sympathetic storm caused by a massive meningeal pain stimulus through blood in the cerebrospinal fluid (CSF) space and an increased ICP seems a plausible explanation for increased catecholamine levels in the CSF and plasma of SAH patients [1, 2, 20].

In line with the results of Kawahara et al. and Levy et al., we hypothesize that a possible initial sympathetic storm with elevated catecholamines may trigger a negative feedback mechanism to cardiogenic centres in the brainstem, supressing the sympathetic influence on the sinus cycle of the heart [17, 21, 22]. Thus, our results of decreased sympathetic autonomic modulation within 24 h after symptom onset may be explained by such feedback mechanisms after the initial catecholamine release. Furthermore, blood induced meningeal affection may trigger the trigemino-cardiac reflex via mechanoreceptors activating the motor nucleus of the vagus nerve. Hence, pain-induced stimulation of the hypothalamus may induce vagal activation and further buffer sympathetic outflow [20, 23, 24].

In conclusion, we assume that the initial meningeal pain stimulus and increasing ICP may induce sympathetic overexcitation and catecholamine release immediately after SAH onset. While brain parenchyma and therefore ANS network structures are not directly affected, various mechanisms, i.e., negative feedback to the vagi and the trigemino-cardiac and vagal reflex may buffer the negative effects of the initial sympathetic storm. This counter regulation seems to depend on clinical severity as we have observed decreasing values of the index of sympatho-vagal balance (RRI-LF/HF-ratios) with higher H&H-scores (Table 2, Fig. 2).

Overall, we cannot rule out an influence of differences in baseline and clinical characteristics or co-medication on our findings. General anaesthesia has previously been associated with decreased HRV [25, 26], and the influence of opioids on the ANS is complex [27]. Furthermore, catecholamines as the predominantly used vasopressors to maintain SBP in critically ill patients induce tachycardia and decrease time-domain measures of HRV, i.e., RRI-SD and RRI-RMSSD [28, 29]. Yet, multivariable regression analyses corrected for confounding variables verified the association of autonomic cardiovascular dysfunction and clinical SAH severity in our cohort, and parameters identified to be associated with initial clinical severity according to H&H, i.e., age, pre-mRS, IVH, and intubation, were not significantly associated with autonomic cardiovascular modulation using multiple logistic regression (Table 3). These statistical tests only represent an approach to estimate the extend of possible confounding parameters and procedures. Although our results suggest that differences in baseline and clinical characteristics or the use of concomitant medication for vasopressor therapy and analgo-sedation may at the most to some extend explain the decrease in HRV observed in all our patients, significance of these results is limited due to the small sample size.

As mentioned above, a reduction of HRV in critically ill patients is associated with a higher risk of cardiovascular events and poor clinical outcome. Yet, the question remains whether autonomic dysfunction in the acute phase of spontaneous SAH may influence clinical outcome. When applying established age-dependant normative values to dichotomize the patient cohort into the two subgroups normal vs impaired autonomic cardiovascular modulation, our results demonstrated significantly higher rates of unfavourable short-term outcome (i.e., mRS at discharge) in patients with autonomic impairment upon testing within 24 h after SAH onset. Although no clear utility for clinical practice can be derived from these findings, our results suggest that the severity of the impairment of autonomic modulation already in the acute phase of SAH may be associated with clinical outcome and possibly a higher risk for severe secondary complications, i.e., arrhythmias, cerebral vasospasm, neurogenic pulmonary edema, or sepsis. Yet, as mRS at discharge may not represent a suitable surrogate measure for functional outcome after SAH, further studies are needed before results may be generalized for clinical utilization.

Despite several strengths and the novelty of our results, some limitations may undermine generality of our study. First, the sample size of our patient group may still have been too small to establish valid predictors for patients at risk of secondary complications after SAH. Defined exclusion criteria only comprised (I) immediate withdrawal of care, (II) lack of assessment of autonomic cardiovascular modulation within 24 h after symptom onset, and (III) cerebral Amyloid angiopathy or trauma as primary cause of SAH to not further minimize our cohort. Thus, the cohort is rather heterogeneous regarding other parameters potentially influencing autonomic cardiovascular modulation, which might limit generalizability of our results. Therefore, some uncertainty remains to interpret the explanatory results of the multivariate analyses considering the imbalance between the small sample size and possible clinical and radiological confounders such as usage of antihypertensive medication or influence of intracranial pressure. Furthermore, we were not able to stratify according to radiological parameters of SAH. Acute occluding hydrocephalus, concomitant parenchymal and intraventricular hemorrhage may vary in their susceptibility in altering autonomic function. We did not correlate autonomic impairment with long-term clinical outcomes after SAH and the mRS at discharge may not be a suitable surrogate parameter for outcome prognostication after SAH. So far, the utility of autonomic monitoring in clinical practice remains unclear. Our findings of an increasing reduction of autonomic cardiovascular modulation with higher H&H-scores during the acute phase after symptom onset encourage follow-up assessments to determine the duration and time course of autonomic dysfunction after SAH. Although possible autonomic perturbations might contribute to or deteriorate secondary complications after SAH, further investigations assessing possible changes of autonomic modulation during the course of in-hospital treatment are needed to clarify whether this approach may reliably predict clinical outcome. Finally, we chose a healthy control group with no known comorbidities and no medication to assure as few confounding factors on the autonomic nervous system modulation as possible. Yet, a second control group—matched for comorbidities and previous medication—would add valuable information in the field of autonomic nervous system research and this aspect should be addressed in follow-up studies.

## Conclusion

In conclusion, within 24 h after SAH, our results show a decline of sympathetic and total autonomic cardiovascular modulation, which might be ascribed to various mechanisms buffering the initial meningeal pain stimulation and ICP elevation. Finally, short-term functional outcome seems to be associated with the severity of autonomic impairment upon hospital admission.

Further studies need to verify these findings to identify whether early autonomic dysfunction may predict complications and influence decision making in SAH patients.

## Supplementary Information

Below is the link to the electronic supplementary material.

Supplementary file1 (DOCX 28 KB)

## Data Availability

Data may be shared by writing to the corresponding author.
