# Supplementary material for: Correlation between clinical severity and extent of autonomic cardiovascular impairment in the acute phase of subarachnoid hemorrhage
Source: J Neurol. 2022 Jun 20;269(10):5541–52. doi: 10.1007/s00415-022-11220-w (PMC9467940; doi:10.1007/s00415-022-11220-w)
Supplement: Supplementary file 1 — Supplementary file1 (DOCX 28 KB) [file 415_2022_11220_MOESM1_ESM.docx]

**Suppl-Table-1 – Antihypertensive premedication and antihypertensive medication during autonomic measurement in 51 patients with spontaneous subarachnoid hemorrhage according to clinical disease severity, either Hunt and Hess score < 3 or ≥ 3**

| Parameter | **SAH Patients**  (n=51) | **Hunt & Hess < 3**  (n=19) | **Hunt & Hess ≥ 3**  (n=32) | ***p*-value** |
| --- | --- | --- | --- | --- |
| **Antihypertensive premedication (n, %)** | 16 (31.4%) | 5 (26.3%) | 11 (34.4%) | 0.549^a^ |
| Mono therapy | 5 (9.8%) | 1 (5.3%) | 4 (12.5%) | 0.527^c^ |
| Dual therapy | 7 (13.7%) | 2 (10.5%) | 5 (18.8%) | 0.699^c^ |
| Three or more antihypertensive drugs | 4 (7.8%) | 2 (10.5%) | 2 (6.3%) | 0.623^c^ |
| **Classes of antihypertensive premedication (n, %)** |  |  |  |  |
| ACE inhibitors or ARBs | 14 (27.5%) | 4 (21.1%) | 10 (31.3%) | 0.527^c^ |
| Calcium channel blockers | 2 (3.9%) | 1 (5.3%) | 1 (3.1%) | 1^c^ |
| Diuretics | 10 (19.6%) | 3 (15.8%) | 7 (21.9%) | 0.725^c^ |
| Beta blockers | 6 (11.8%) | 2 (10.5%) | 4 (12.5%) | 1^c^ |
| **Antihypertensive therapy during measurement (n, %)** | 9 (17.6%) | 5 (26.3%) | 4 (12.5%) | 0.266^c^ |
| Alpha-1-adrenoceptor antagonist | 7 (13.7%) | 4 (21.1%) | 3 (9.4%) | 0.402^c^ |
| Alpha-2-adrenoceptor agonist | 1 (2.0%) | 1 (5.3%) | 0 (0.0%) | 0.373^c^ |
| Dihydralazine | 1 (2.0%) | 0 (0.0%) | 1 (3.1%) | 1^c^ |
| others | 0 (0.0%) | 0 (0.0%) | 0 (0.0%) | 1^c^ |

SAH, subarachnoid hemorrhage; n, number; ACE, Angiontensin-converting-enzyme; ARB, angiotensin II receptor blockers.

^a^ p-values derived from Chi-square-test of association

^b^ significant differences between subgroups

^c^ p-values derived from Fisher exact probability test.
